# Low dose of 2-deoxy-D-glucose kills acute lymphoblastic leukemia cells and reverses glucocorticoid resistance via N-linked glycosylation inhibition under normoxia

**DOI:** 10.18632/oncotarget.16046

**Published:** 2017-03-09

**Authors:** Ling Gu, Zhihui Yi, Yanle Zhang, Zhigui Ma, Yiping Zhu, Ju Gao

**Affiliations:** ^1^ Laboratory of Hematology/Oncology, Department of Pediatric Hematology/Oncology, Key Laboratory of Birth Defects and Related Diseases of Women and Children, Ministry of Education, West China Second University Hospital, Sichuan University, Chengdu, China; ^2^ Department of Gastroenterology, West China University Hospital, Sichuan University, Chengdu, China

**Keywords:** 2-deoxy-D-glucose, glycolysis, glucocorticoid resistance, acute lymphoblastic leukemia, N-Linked glycosylation

## Abstract

Recent studies showed that 2-deoxy-D-glucose (2-DG), a glucose analog with dual activity of inhibiting glycolysis and N-linked glycosylation, can be selectively taken up by cancer cells and be used as a potential chemo- and radio-sensitizer. Meanwhile, 2-DG can kill cancer cells under normoxia. However, its efficacy is limited by the high-dose induced systemic toxicity. Here, we showed that low-dose 2-DG could be used as a single agent to kill acute lymphoblastic leukemia (ALL) cells, and as a GC sensitizer to overcome GC resistance under normoxia. Addition of exogenous mannose, a sugar essential for N-linked glycosylation, rescued 2-DG-treated ALL cells, indicating that inhibition of N-linked glycosylation and induction of endoplasmic reticulum stress is the main mechanism for 2-DG to induce cell death and reverse GC resistance in ALL cells. These data provides new insight into the molecular mechanisms involved in GC resistance. More important, it indicates that 2-DG might be the promising drug for designing novel high efficiency and low toxic protocol for ALL patients.

## INTRODUCTION

Acute lymphoblastic leukemia (ALL) represents the most common cancer in children. Recently, ALL is highlighted as a cancer success story for pediatric patients, with a 5-year event free survival higher than 80% in most current treatment protocols [1, 2]. However, still 15–20% of the patients relapse, with a significantly worse prognosis [3]. A hallmark of relapses is acquired resistance to multiple chemotherapeutic agents, particularly glucocorticoids (GCs) [4]. Novel strategies are needed to overcome chemoresistance, especially GC resistance, and improve outcome. Moreover, along with the high survival rate, more and more patients are bearing treatment-related late effects such as secondary malignancy, cardiotoxicity, obesity, endocrine abnormalities, reproductive changes, neurocognitive deficits and psychosocial effects [5, 6]. Therefore, there is an urgent need for novel treatment options to enhance chemosensitivity and overcome GC resistance, which will help to reduce the intensity of chemotherapy and then minimize the toxicity of traditional chemotherapy.

Reprogramming of energy metabolism is a hallmark of cancer and plays a key role in chemoresistance in different cancers [7–9]. Cancer cells become dependent on aerobic glycolysis, making high glucose uptake essential [10]. Based on this mechanism, 2-deoxy-2-[fluorine-18] fluoro-D-glucose emission tomography (^18^F-FDG-PET) is used to detect and monitor cancers non-invasively by visualizing whole body glucose uptake [11, 12]. PET/CT with ^18^F-FDG is a powerful tool for the diagnosis, staging, and follow-up of patients with solid or hematologic malignancies [13–15]. More important, in addition to selective uptake by cancer cell, 2-Deoxy-D-glucose (2-DG) inhibits the proliferation of cancer cells through inhibition of glycolysis and N-linked glycosylation [16]. Under normoxia, 2-DG at a low-concentration is not toxic in most tested carcinomas, however, it may represent a useful radio- and chemo-sensitizing drug [17, 18]. Our previous study showed that 2-DG could inhibit glucose uptake and reverse GC resistance in Burkitt lymphoma Raji cells [19].

In this study, we found that low dose of 2-DG (1 mM) could induce cell death and overcome GC resistance in ALL under normoxia. Interestingly, these effects were achieved mainly by inhibiting N-Linked glycosylation rather than inhibiting glycolysis. Therefore, 2-DG might be used as a novel treatment option to overcome GC resistance and minimize the toxicity of traditional chemotherapy.

## RESULTS

### Low-dose 2-DG reduces cell viability in ALL cells under normoxia

**Figure 1:**
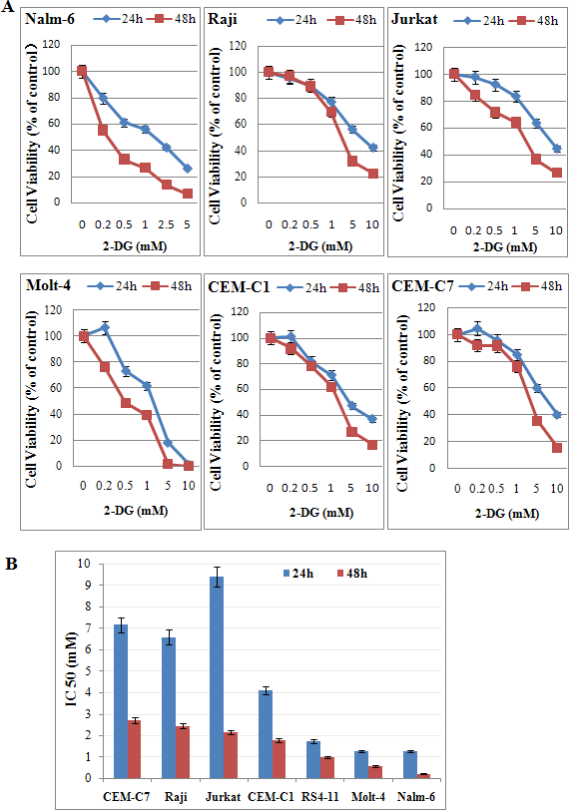
Low-dose 2-DG reduces cell viability in ALL cells under normoxia (**A**) ALL and Raji cells were cultured with increasing concentrations of 2-DG (ranging from 0.2 to 10 mM) for 24 h and 48 h. The viability rates of the cells were evaluated with an MTT assay. The experiments were performed in triplicate. (**B**) IC50 values calculated by a decrease of 50% in cell viability rate compared to that of the control cell.

Previously, it was reported that 2-DG does not kill most cancer cells under normoxic condition [17]. To evaluate the efficacy of 2-DG under normoxic condition, ALL and Raji cells (B-lineage GC resistant lymphoid cells) were treated with increasing concentrations of 2-DG for 24 h and 48 h, followed by assessment of cell viability using 3-(4,5-dimethylthiazol-2-yl)-2,5-diphenyltetrazolium bromide (MTT) assays. As shown in Figure 1, 2-DG induced cell viability inhibition under normoxic condition in all tested cells in a dose and time dependent manner (Figure 1A) with an IC50 (concentration that inhibits 50% ) ranging from 0.22 mM in the most sensitive cell line Nalm-6 to 2.70 mM in CEM-C7-14 at 48 h (Figure 1B).

### Low-dose 2-DG induces cell death and G_0_/G_1_ phase arrest in ALL cells

**Figure 2:**
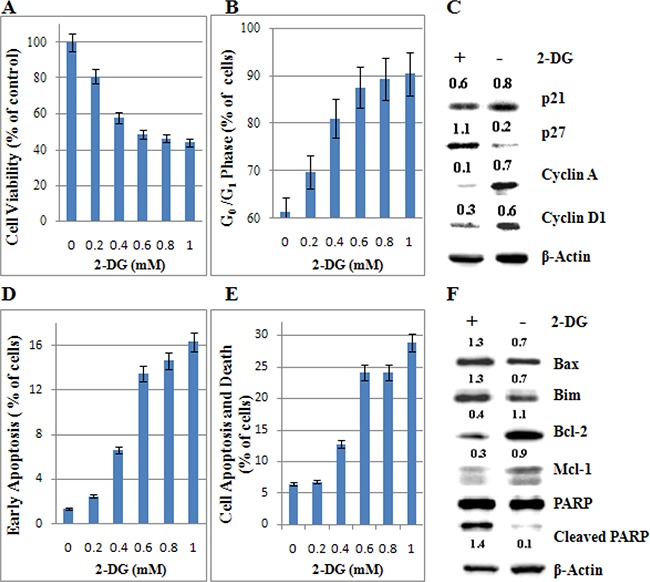
Low-dose 2-DG induces cell death and G_0_/G_1_ phase arrest in ALL cells under normoxia (**A**) Molt-4 cells were incubated with increasing concentrations of 2-DG (ranging from 0.2 to 1 mM) for 48 h. The viability rates of the cells were evaluated with an MTT assay. The experiments were performed in triplicate. (**B**) The cell cycle was analyzed by PI staining using flow cytometry. For all experiments, values of triplicate experiments are shown as the mean ± SD. (**C**) After 48 h exposure to 2-DG, cells were lysed and extracts were analyzed by western blotting for cell cycle regulatory proteins. β-Actin was used as an internal control. The experiments were performed in triplicate. (**D**) The early stage of apoptosis was detected by Annexin V-FLUOS/PI staining (*Annexin V* *positive*/*PI* *negative*). (**E**) The rate of cell apoptosis and death was detected by Annexin V-FLUOS/PI staining (*Annexin V positive*). (**F**) The levels of apoptotic associated proteins were detected by western blotting. β-Actin was used as an internal control. The experiments were performed in triplicate. +: positive; −: negative.

T-ALL always has a poor prognosis [20]. Molt-4, a GC resistant cell line, established from the peripheral blood of a 19-year-old man with T-ALL in relapse [21], is sensitive to 2-DG treatment (Figure 1). Therefore, we selected Molt-4 cell line to investigate the anti-leukemic mechanism of 2-DG. Along with the increasing concentration of 2-DG (0~1 mM), cell viability decreased from 100% to 44% (Figure 2A), G_0_/G_1_ phase increased from 61% to 90% (Figure 2B), Annexin V positive and propidium iodide (PI) negative (cell early apoptosis) rate increased from 1% to 16% (Figure 2D), cell apoptosis and death (Annexin V positive) rate increased from 7% to 29% at 48 h (Figure 2E). To evaluate the molecular basis underlying cell cycle arrest and cell apoptosis, we investigated the expression of cell cycle and apoptosis regulatory proteins. As shown in Figure 2C, after 48 h treatment, 1 mM 2-DG induced the expression of CDK inhibitors p27and reduced Cyclin A and Cyclin D1 levels. Figure 2F showed that, after 48 h treatment of 1 mM 2-DG, Bim and Bax increased clearly, Bcl-2 and Mcl-1 decreased dominantly and poly ADP-ribose polymerase (PARP) was cleaved, which indicated that 2-DG induced apoptosis through the mitochondrial pathway.

### Low-dose 2-DG overcomes GC resistance in ALL cells

**Figure 3:**
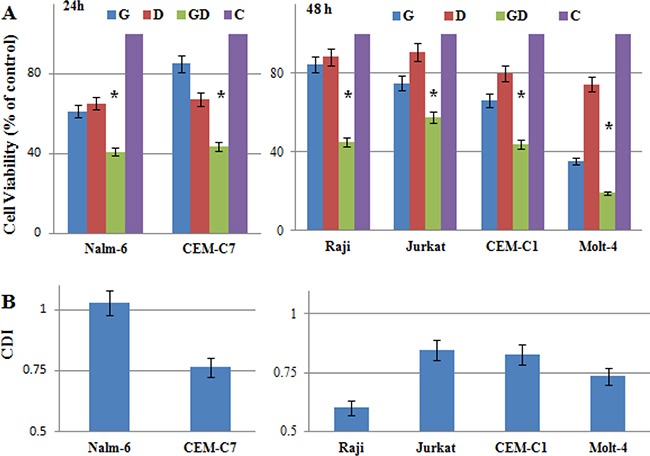
Low-dose 2-DG overcomes GC resistance in ALL cells (**A**) ALL and Raji cells were incubated for 24 h (GC sensitive) and 48 h (GC resistance) with 2-DG (1 mM) and/or Dex (1 μM). The viability rates of the cells were evaluated with an MTT assay. The experiments were performed in triplicate. For all experiments, values are presented as the mean ± SD (*n* = 3) *p < 0.01 versus the control group, Dex group, or 2-DG group. (**B**) CDI was used to analyze effects of drug combinations. CDI value < 1, = 1 or > 1 indicates that the drugs are synergistic, additive or antagonistic, respectively. CDI value < 0.75 indicates that the drugs are significantly synergistic. Values are the results of 3 determinations. G, 2-DG group; D, Dex group; GD, 2-DG+Dex group and C, control group.

More and more studies reported that increased aerobic glycolysis is a hallmark of cancer and plays a role in chemoresistance in different cancer cells [7–9, 22, 23]. To directly demonstrate if 2-DG treatment can reverse GC resistance, we incubated ALL and Raji cells with 1 mM 2-DG and/or 1 μM dexamethasone (Dex) for 24 h and 48 h. 2-DG combined with Dex inhibited the viability of GC resistant cells (Raji, Jurkat, CEM-C1-15, Molt-4) synergistically (Figure 3A). The coefficient of drug interaction (CDI) was ranging from 0.61 in the most sensitive cell line Raji to 0.85 in Jurkat at 48 h (Figure 3B), indicating that combined treatment produced a significant synergistic effect in Raji and Molt-4 cells. In GC sensitive ALL cells, 2-DG and Dex inhibited the growth of CEM-C7-14 synergistically, the CDI was 0.76 at 24 h (Figure 3A and 3B). Nalm-6 cells are quite sensitive to both 2-DG and Dex, combined treatment only showed additivity effect, the CDI was 1.01 at 24 h (Figure 3A and 3B).

**Figure 4:**
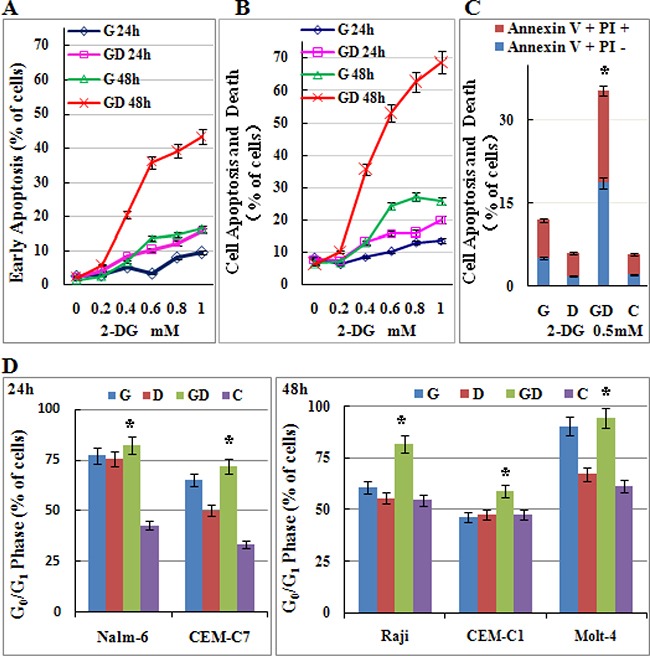
Low-dose 2-DG treatment sensitizes ALL cells to GC treatment by inducing apoptosis and G_0_/G_1_ phase arrest (**A**) Molt-4 cells were incubated with increasing concentrations of 2-DG (ranging from 0.2 to 1 mM) and/or Dex (1 μM) for 24 h and 48 h. The early stage of apoptosis was detected by Annexin V-FLUOS/PI staining (*Annexin V positive*/*PI* *negative*). For all experiments, values of triplicate experiments are shown as the mean ± SD. (**B**) The rate of cell apoptosis and death was detected by Annexin V-FLUOS/PI staining (*Annexin V positive*). (**C**) Molt-4 cells were incubated with 2-DG (0.5 mM) and/or Dex (1 μM) for 48 h. The rate of cell apoptosis and death was detected by Annexin V-FLUOS/PI staining (*Annexin V positive*). *p < 0.01 versus the control group, Dex group or 2-DG group. (**D**) ALL cells were incubated for 24 h and 48 h with 2-DG (1 mM) and/or Dex (1 μM). The cell cycle was analyzed by PI staining. *p < 0.01 versus the control group. G, 2-DG group; D, Dex group; GD, 2-DG+Dex group and C, control group.

GCs exert antileukemic activity through inducing both apoptosis and cell-cycle arrest. To further make sure that 2-DG can restore the antileukemic effect of GC, we incubated Molt-4 cells with increasing concentrations of 2-DG (0~1 mM) and/or 1 μM Dex. As shown in Figure 4A, 0.2 mM 2-DG combined with 1 μM Dex did not induce obviously cell apoptosis. After the concentration of 2-DG was elevated to 0.4 mM, combined treatment induced cell apoptosis and cell death synergistically in a dose dependent manner (Figure 4A and 4B). 0.5 mM 2-DG combined with 1 μM Dex induced the early apoptotic rate to 19%, and the cell apoptosis and death rate to 35% (Figure 4C). When 2-DG was elevated to 1 mM, the early apoptotic rate elevated to 43% and the cell apoptosis and death rate elevated to 69% in combined group (Figure 4A and 4B). Combined treatment induced cell cycle arrested in G_0_/G_1_ phase in all detected cell lines (Figure 4D). However, 2-DG used alone induced G_0_/G_1_ arrest greatly. Combined treatment showed different effects on cell cycle, synergistic, additive or even antagonistic in different cell lines. These results indicated that a very low dose of 2-DG (0.4 mM) could restore the Dex sensitivity in Molt-4 cells by inducing cell apoptosis.

### The glycolytic phenotype dose not correlate with the sensitivity to 2-DG in ALL

**Figure 5:**
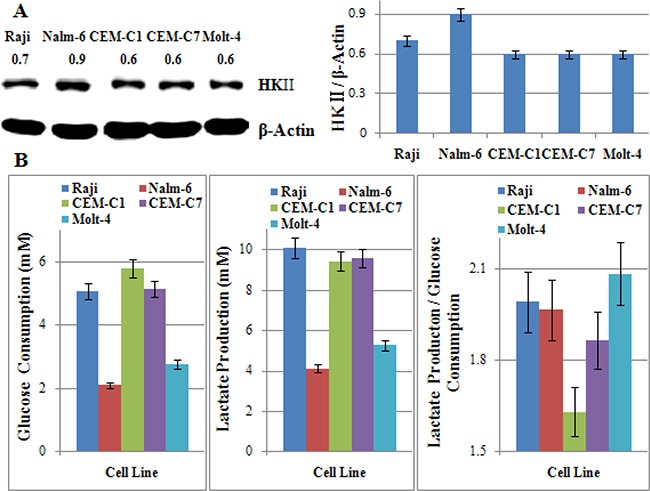
The glycolytic phenotype dose not correlate with the sensitivity to 2-DG in ALL cells (**A**) Cells were lysed and extracts were analyzed by western blotting for HKII. β-Actin was used as an internal control. Bar graphs show the ratio of HK to β-Actin. For all experiments, values of triplicate experiments are shown as the mean ± SD. (**B**) Glucose consumption was measured with the Glucose (HK) Assay Kit. The concentration of lactate was analyzed using a Lactate Assay Kit. The ratio of lactate production to glucose consumption showed the glycolytic phenotype of cells. For all experiments, values of triplicate experiments are shown as the mean ± SD.

2-DG is a most frequently used glycolytic inhibitor that induces growth arrest and cell death by inhibiting the activity of the key glycolytic enzyme hexokinase (HK) and phosphoglucoisomerase [16–18]. HKII, a key enzyme involved in catalyzing the first committed step of glucose metabolism, has been recognized as an oncogenic kinase, as it is over-expressed in many cancers and contribute to tumor initiation progression, and resistance to therapy [24–26]. In our study, we found that all tested cells over-expressed HKII. However, there was no obvious different in the expression of HKII in different cell lines except Nalm-6 (Figure 5A). To determine the glycolytic phenotype of those cells, we tested the glucose consumption and lactate production, and then calculated the ratio of lactate production to glucose consumption. The increase in the ratio of lactate production to glucose consumption in the presence of oxygen showed the increase in aerobic glycolysis in ALL cells. According to Figure 5B, Figure 1B, and Figure 3B, the sensitivity to 2-DG alone or in combination with Dex was not consistent to the glycolytic phenotype in ALL and Raji cells. That is to say, the expression of HKII and the glycolytic phenotype did not affect the cytotoxic effect of 2-DG under normoxic condition in ALL cells.

### Mannose rescues 2-DG-induced cell viability inhibition and GC sensitization

**Figure 6:**
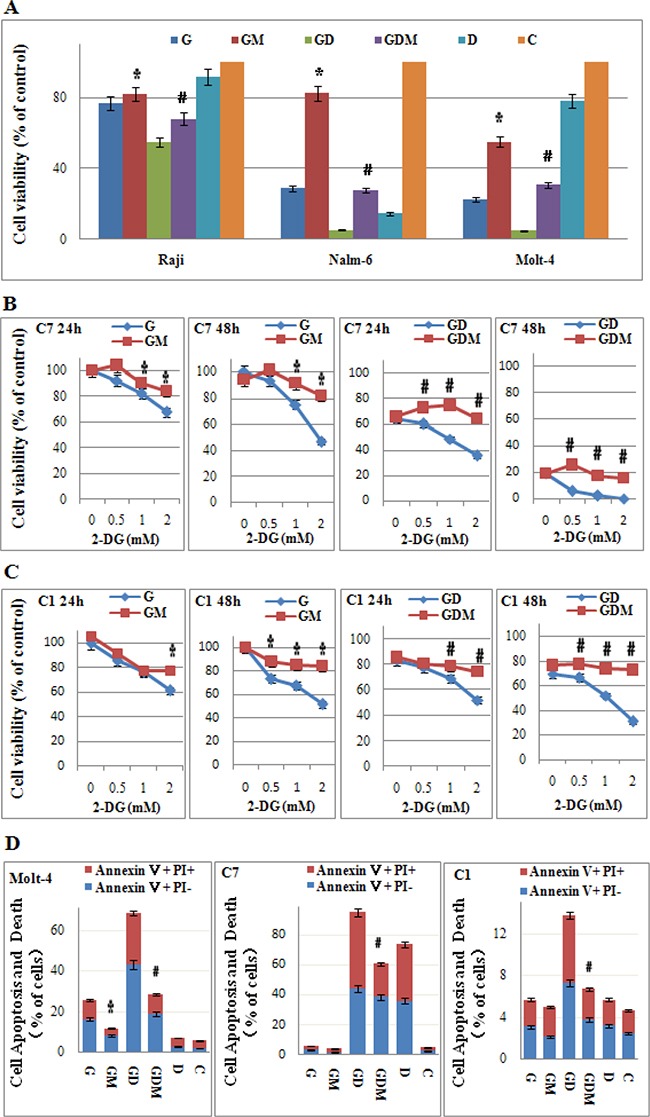
Mannose rescues 2-DG-induced cell viability inhibition, cell death and GC sensitization (**A**) ALL cells were incubated with 2-DG (1 mM) and/or Dex (1 μM) and/or mannose (2 mM) for 24 h and 48 h. The viability rates of the cells were evaluated with an MTT assay. *p < 0.01 versus the 2-DG group. ^#^*p <* 0.01 versus the 2-DG+Dex group. (**B**) CEM-C7-14 cells were incubated with increasing concentrations of 2-DG (ranging from 0.5 to 2 mM) and/or Dex (1 μM) and/or mannose (2 mM) for 24 h and 48 h. The viability rates of the cells were evaluated with an MTT assay. *p < 0.01 versus the 2-DG group. ^#^*p <* 0.01 versus the 2-DG+Dex group. C7: CEM-C7-14. (**C**) CEM-C1-15 cells were incubated with increasing concentrations of 2-DG (ranging from 0.5 to 2 mM) and/or Dex (1 μM) and/or mannose (2 mM) for 24 h and 48 h. The viability rates of the cells were evaluated with an MTT assay. *p < 0.01 versus the 2-DG group. ^#^*p <* 0.01 versus the 2-DG+Dex group. C1: CEM-C1-15. (**D**) The rate of cell apoptosis and death was detected by Annexin V-FLUOS/PI staining (*Annexin V positive*) after 48 h treatment (2-DG 1 mM) in Molt-4, CEM-C7-14 and CEM-C1-15 cells. *p < 0.01 versus the 2-DG group. ^#^*p <* 0.01 versus the 2-DG+Dex group. G, 2-DG group; GM, 2-DG+mannose group; GD, 2-DG+Dex group; GDM, 2-DG+Dex+mannose group; D, Dex group and C, control group.

2-DG has a dual activity of inhibiting glycolysis and N-linked glycosylation [27]. Increasing evidence indicates that interfering with N-linked glycosylation is the key mechanism in eliciting tumor cell death under normoxia [28–30]. To further explore the molecular mechanism, we co-treated cells with mannose to rescued N-linked glycosylation. 2 mM mannose did not affect the cell proliferation or viability (data not shown) under normoxia. Figure 6A showed that mannose rescued the cells viability from 29% to 83% in the most effective cell line Nalm-6, and 77% to 82% in Raji cells after 48 h treatment when 2-DG used alone. Along with the increasing dosage of 2-DG (0~2 mM), cell viability decreased from 100% to 46% or 52% in CEM-C7-14 and CEM-C1-15 respectively, mannose restored the cell viability to higher than 80% (Figure 6B and 6C). The cell apoptosis and death induced by 2-DG was mostly rescued by mannose (Figure 6D). Therefore, inhibition of N-linked glycosylation was shown to be the main mechanism of cytotoxicity induced by 2-DG under normoxic condition in ALL cells. In combined group, mannose restored the cell viability to the baseline level (Dex used alone) in GC sensitive cell lines Nalm-6 and CEM-C7-14, and GC resistant cell line CEM-C1-15 after 48 h treatment (Figure 6A–6C). In Raji and Molt-4 cells, mannose partly inhibited the synergistic effect induced by 2-DG and Dex (Figure 6A). Similarly, the cell apoptosis and death was fully rescued to the baseline level by mannose in CEM-C1-15 and CEM-C7-14 cells (Figure 6D). In Molt-4 cells, consistent to the effect on cell viability, mannose rescued the cells from death partly. These findings implicated N-linked glycosylation inhibition as a main mechanism for GC resensitization by 2-DG.

### Induction of ER stress leads to 2-DG induced cell death and GC sensitization

**Figure 7:**
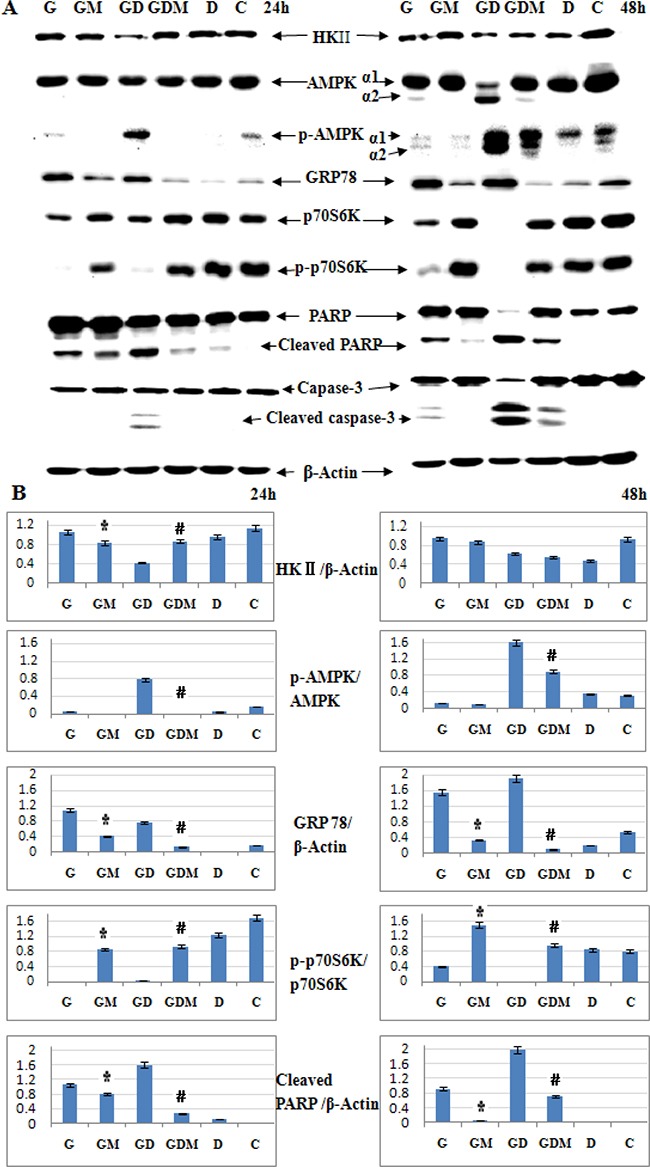
Induction of ER stress leads to 2-DG induced cell death and GC sensitization (**A**) Western blot analysis of HKII, AMPKα, p-AMPKα (Thr172), GRP78, p70S6K, p-p70S6K (Thr421/Ser424) and cell apoptosis associated proteins in Molt-4 cells after 48 h exposure to 2-DG, Dex, mannose, alone or in combination. β-Actin was used as an internal control. (**B**) Bar graphs show the ratio of protein to β-Actin and phospho-protein to total protein. For all experiments, values of triplicate experiments are shown as the mean ± SD. *p < 0.01 versus the 2-DG group. ^#^*p <* 0.01 versus the 2-DG+Dex group. G, 2-DG group; GM, 2-DG+mannose group; GD, 2-DG+Dex group; GDM, 2-DG+Dex+mannose group; D, Dex group and C, control group.

Under normoxia, 2-DG induces cell death by interfering with N-linked glycosylation, which leads to accumulation of misfolded proteins and an endoplasmic reticulum (ER) stress response [28]. To further explore the molecular mechanisms by which 2-DG induces cell death and GC sensitivity in ALL, we examined the expression of HKII and critical signaling proteins associated with energy metabolism in general (AMP-activated protein kinase, AMPKα and p-AMPKα), ER stress marker (glucose-regulated protein 78, GRP78), p70S6 kinase (p70S6K), p- p70S6K and cell apoptosis in Molt-4 cells. As shown in Figure 7, after 48 h treatment, 1 mM 2-DG did not inhibit the expression of HKII. Meanwhile, p-AMPK (Thr172) was not induced. Interestingly, after 24 h treatment, 2-DG decreased the expression of p70S6K and p-p70S6K (Thr421/Ser424) obviously (Figure 7A and 7B). On the contrary to p-p70S6K, GRP78 was induced dominantly (Figure 7A and 7B). After 48 h treatment, cell apoptosis was triggered by 2-DG, which was detected by cleaved PARP and cleaved caspase-3. Mannose completely restored the expression of GRP78, p-p70S6K, PARP and caspase-3. These data indicated that induction of ER stress led to 2-DG induced cell death.

Different to 2-DG treatment alone, combined with Dex inhibited HKII expression at 24 h, recovered a part at 48 h (Figure 7A and 7B). Notably, combined treatment decreased AMPKα1 dominantly with significantly inducing of AMPKα2 after 48 h treatment. And the expression of p-AMPKα1(Thr172) was induced after 24 h, both p-AMPKα1 (Thr172) and p-AMPKα2 (Thr172) were induced dominantly after 48 h (Figure 7A and 7B). AMPK has been proposed as a physiological cellular energy sensor [31]. The induction of p-AMPKα (Thr172) indicates combined treatment led to a decrease in intracellular adenosine triphosphate (ATP) concentration. Moreover, mannose did not fully rescue the expression of p-AMPKα (Thr172) to the baseline level (Dex used alone) (Figure 7A and 7B). That means the energy inhibited by combined treatment would not be fully rescued by mannose. Similar to 2-DG treatment alone, GRP78 was induced by combined treatment and fully restored by mannose. The expression of p70S6K and p-p70S6K (Thr421/Ser424) decreased obviously. These proteins were almost fully restored by mannose. Combined treatment cleaved PARP and caspase-3 clearly at 48 h, mannose restored the expression of these proteins partly (Figure 7A). That means, in 2-DG sensitive cell line, inhibition of glycolysis may help to overcome GC resistance under normoxia. Taken together, these data suggest that induction of ER stress is the predominant mechanism responsible for 2-DG to overcome GC resistance.

## DISCUSSION

Although remarkable progress has been made in the treatment of ALL in the past two decades, with the proportion of patients surviving for 5 years approaching and even exceeding 90% in many developed countries [32], still 15~20% of the children relapse with an acquired chemoresistance, and more and more patients are suffering treatment-related late effects [3, 5, 6]. Further improvement in the treatment outcome and quality of life will require novel and optimizing treatment to maximize the effect of killing leukemia cells and minimize the toxicity of traditional chemotherapy. The novel drugs are needed to target cancer cells, sensitize chemotherapy and reverse chemoresistance.

In our previous study, we found that inhibition of p70S6K/glycolysis signaling pathway plays an essential role in reversing GC resistance in Burkitt lymphoma Raji cells [19]. Emerging evidence indicates that cancer is primarily a metabolic disease involving disturbances in energy production [33]. The metabolic shift towards aerobic glycolysis with reprogramming of mitochondrial oxidative phosphorylation, regardless of oxygen availability, is a phenomenon known as the Warburg effect [10].This alternative energy metabolism strategy used by tumor cells makes it a potential targetable pathway for cancer diagnosis and therapy. One of the most frequently used antiglycolytic agents is 2-DG, a non-metabolizable sugar analog, which is selectively taken up by cancer cells, phosphorylated by HK and subsequently inhibits ATP generated *via* the glycolytic pathway [16]. Our study showed 2-DG inhibited glucose uptake and reversed GC resistance in Burkitt lymphoma Raji cells [19]. Therefore, 2-DG might be the potent drug we are looking for. Recently, it has been shown that like those solid tumor cells, leukemia cells also exhibit increased glycolytic rate even in the presense of sufficient oxgen [34].

As expected, 2-DG inhibited the growth of ALL cells under normoxic conditions in a dose and time dependent manner by inducing cell death and G_0_/G_1_ cell cycle arrest. It is worthy to note that, T-ALL cells were sensitive to 2-DG just like B-ALL. Moreover, the tested T-ALL cell lines, CEM-C7-14, CEM-C1-15, Jurkat, Molt-4, were all established from T-ALL patients at relapse. T-ALL patients often have high-risk clinical features such as older patient age, high leukocyte count, or other indications of high tumor burden, and central nervous system involvement is not uncommon [35]. Therefore, patients with T-ALL have a poorer prognosis than patients with B-ALL. Nowadays, intensified chemotherapy protocols have increased the cure rate to approximately 75% in pediatric patients [36]. Nevertheless, patients with T-ALL remain at increased risk for remission induction failure, early relapse, isolated CNS relapse and late effects caused by the high-dose chemotherapy [35, 36]. Our results indicated that 2-DG is a potent drug to induce cell death of T-ALL cells and might help to improve the prognosis for T-ALL patients.

Under hypoxic conditions where glycolysis is the only source of energy, 2-DG severely depletes ATP eventually leading to massive cell death [16, 17]. In most tumor cell lines, 2-DG always induces cell death under hypoxia conditions and it is toxic only in select tumor cell lines growing under normoxic conditions [17, 18]. However, under nomoxia, high concentrations (~20 mM) of 2-DG were typically used to inhibit the glycolytic metabolism in cancer cells [37, 38]. Therefore, its efficacy is limited by the systemic toxicity [37, 39]. In our study, 0.4 mM 2-DG, a quite low dosage, could induce cell death in T-ALL Molt-4 cell line under normoxia. And the IC 50 of all those tested cell lines is ranged from 0.22 to 2.27 mM. Notably, the sensitivity to 2-DG did not correlate with the glycolytic phenotype. In Molt-4 cells, according to the expression of HKII, AMPK and p-AMPK (Thr172), 1 mM 2-DG had almost no effect on the energy metabolism after 48 h treatment. So, the antileukemic efficiency of 2-DG on ALL cells cannot be explained by glycolytic inhibition. 2-DG kills select tumor cells under normoxic conditions through inhibiting N-linked glycosylation and inducing ER stress mediated apoptosis [28–30]. In our study, 2-DG alone induced the expression of GRP78 clearly. GRP78, a predominant ER chaperone, promotes cell proliferation, survival and metastasis. ER stress induces GRP78 expression and promotes an interaction between GRP78 and AKT, which in turn suppresses Ser473 phosphorylation of AKT and thereby modulates substrate, including AKT/mTOR/p70S6K pathway, specificity [40]. As expected, low-dose 2-DG decreased the expression of p70S6K and p-p70S6K (Thr421/Ser424) obviously with the increase of GRP78. Therefore, mannose, a sugar essential for N-linked glycosylation, was added to 2-DG-treated cells to inhibit ER stress. In our study, 2 mM mannose almost fully restored the expression of all those proteins and cell viability in ALL cells. Our results confirmed that low-dose 2-DG kills ALL cells through inhibiting N-linked glycosylation and inducing ER stress mediated apoptosis.

GCs induce cell cycle arrest and apoptosis in ALL cells and therefore constitute a central component in the treatment of lymphoid malignancies. GC resistance is a well recognized feature of poor prognosis in the treatment of childhood ALL and several mechanisms have been suggested [41]. Methods to overcome GC resistance are still lacking in clinic. Targeting glycolysis pathway may be a particular promising strategy to reverse GC resistance in pediatric ALL [42, 43]. 2-DG increased the sensitivity of NHL cells to methylprednisolone via down-regulation of hypoxia-inducible factor 1-alpha and c-Myc [23]. Our results reconfirmed that, low dose of 2-DG (1 mM) combined with Dex restored the sensitivity of GC and showed synergistic killing effects in T and B lineage ALL cells. Similar to 2-DG treatment alone, combined treatment increased GRP78 and cleaved PARP and caspase-3 obviously by inducing ER stress. Moreover, mannose fully rescued cell death and viability induced by combined treatment in CEM-C1-15 and CEM-C7-14 cell lines. Even in 2-DG sensitive Molt-4 cell line, mannose could partly rescue the antileukemic effect induced by combined treatment. That is to say, N-linked glycosylation inhibition is the key mechanism for GC resensitization by 2-DG rather than glycolysis inhibition. We know that normal tissue toxicity is one of the major limiting factors in cancer therapy. Farooque *et al*. reviewed studies of 2-DG *in vitro*, *in vivo* and in clinical trial, and concluded that normal cells and tissues are spared or protected against radiation or chemotherapy damage by 2-DG [44]. Moreover, clinical trials showed that 2-DG is safe to use at 250 mg/kg body weight (~1.5 mM) given weekly for 7 weeks [45]. Thus, 1 mM 2-DG can be used safely in ALL patients in clinic.

Taken together, our study highlights the potential of 2-DG as a low-toxic drug to selectively kill ALL cells and overcome GC resistance. Inhibition of the N-linked glycosylation and induction of ER stress plays an essential role in inducing cell death and reversing GC resistance in ALL cells, which provides new insight into the molecular mechanisms involved in GC resistance. Addition of 2-DG or its analog to the current treatment protocol for ALL patients may predict an even better long time survival and cure rate especially for those refractory and relapsed patients.

## MATERIALS AND METHODS

### Cell lines and culture conditions

T-ALL cell lines, Molt-4 (GC resistance) and Jurkat (GC resistance) were kindly provided by Dr. Morris (St. Jude children's Research Hospital); CEM-C1-15 (GC resistance) and CEM-C7-14(GC sensitive) were isolated from a patient with ALL [46] and kindly provided by Dr. Thompson (The University of Texas Medical Branch). B-ALL cell line, Nalm-6 (GC sensitive), RS4:11 (GC sensitive) and Burkitt lymphoma cell line Raji (B-lineage, GC resistance) were purchased from Shanghai Institute Cell Resources Bank. All cell lines were maintained in RPMI 1640 (Hyclone, Logan, UT, USA) supplemented with 10% fetal bovine serum (FBS; Hyclone), at 37°C in a humidified 5% CO_2_ in-air atmosphere.

### Reagents and antibodies

2-DG (Sigma, St. Louis, MO, USA) was dissolved in phosphate-buffered saline (PBS) and used at the concentration of 0~10 mM. Dex (Sigma) was dissolved in ethanol and used at the concentration of 1 μM, the final concentration of ethanol in the medium was 0.01%, at which cell growth was not obviously altered. Mannose (sigma) were dissolved in PBS and used at the concentration of 2 mM, at which cell growth was not obviously affected. PI and MTT were purchased from Sigma. The Annexin V-PI Kit was purchased from *Roche* (Mannheim, Germany). Antibodies to HKII, p70S6K, p-p70S6K (Thr421/Ser424), AMPK, p-AMPK (Thr172), GRP78, Cyclin D1, p27, PARP, Caspase-3, Bax, Mcl-1, Bim and Bcl-2 were purchased from Cell Signaling Technology (Beverly, MA, USA). The antibody for p21 was purchased from BD Bioscience (San Jose, CA, USA). Antibodies to Cyclin A, horseradish peroxidase (HRP)–conjugated donkey anti-rabbit antibody and HRP-conjugated sheep anti-mouse antibodies were obtained from Santa Cruz Biotech (Santa Cruz, CA, USA). The β-Actin antibody was obtained from Kangchen Bio-Tech (Shanghai, China).

### Cell treatment

Logarithmically growing cells were harvested and replaced in 96-well sterile plastic culture plates and 25 cm^2^ flasks (Corning Inc.), to which various concentrations of 2-DG or 1 μM Dex, specifically 1 mM 2-DG (2-DG group), 1 μM Dex (Dex group), 1 mM 2-DG plus 1 μM Dex (2-DG+Dex group) and 0.01% ethanol (Control group), were added respectively. At the end of the incubation period, cells were transferred to sterile centrifuge tubes, pelleted by centrifugation at 400 g at room temperature for 5 min, and prepared for analysis as described below.

### Cell viability assay

MTT assays were performed as described previously [19]. Briefly, cells were seeded in 96-well plates (200,000/ml) and incubated for 24 or 48 h. Next, 0.5 mM MTT (final concentration) was added to each well for 4 h at 37°C. Then, solubilization buffer (10% SDS in 0.01 M HCl) was added to each well, and the plates were further incubated for 24 h at 37°C. The spectrophotometric absorbance was measured at 570 nm (reference 690 nm) using a multi-plate reader (Multiskan Spectrum, Thermo Electron Co., Waltham, MA, USA). Values were obtained by comparing the experimental cells with their respective controls. Mean values were calculated from triplicate cultures. The CDI was used to analyze the effects of drug combinations. The CDI is calculated as follows: CDI = AB/(A × B). According to the absorbance of each group, AB is the ratio of the combination groups to control group; A or B is the ratio of the single agent group to control group. Thus, a CDI value < 1, = 1 or > 1 indicates that the drugs are synergistic, additive or antagonistic, respectively. CDI < 0.75 indicates that the drugs are significantly synergistic.

### Cell cycle analysis

For each analysis, 10^6^ cells were harvested 48 h after treatment and fixed overnight in 70% ethanol at 4°C. Cells were then washed and stained with 5 μg/ml PI in the presence of DNAse-free RNAse (Sigma). After 30 min at room temperature, the cells were analyzed via flow cytometry (Cytomics FC 500 and CXP & Multicycle software, Beckman Coulter Inc., Miami, FL, USA), acquiring 30,000 events.

### Apoptosis assay

The samples were washed with PBS twice and stained with Annexin V-FLUOS and PI using Annexin-V-FLUOS staining kit (Roche) according to the manufacturer's protocol. The percentage of Annexin-V positive and PI negative cells was determined by flow cytometry (Cytomics FC 500 and CXP software, Beckman Coulter), as the percentage of cells in the early stage of apoptosis, the percentage of Annexin-Vpositive and PI positive was recorded as the percentage of late apoptosis and necrosis. The percentage of Annexin-Vpositive was recorded as the percentage of cell apoptosis and death.

### Glucose consumption assay

Glucose consumption was measured with a Glucose (HK) Assay Kit (Sigma). Briefly, 2 × 10^6^ cells were grown in 10 ml RPMI containing 2 g/L glucose. After 48 h, the medium was collected by centrifugation to remove the cells. Medium from each condition was incubated for 30 min with the glucose assay reagent. Spectrophotometric absorbance was measured at 340 nm using a multi-plate reader (Multiskan Spectrum). Values were obtained by comparing with a glucose standard solution.

### Lactate concentration assay

The concentration of lactate, the final metabolic product of the glycolytic pathway, was analyzed using a Lactate Assay Kit (Jiancheng, Nanjing, China). Briefly, 2 × 10^6^ cells were grown in 10 ml RPMI containing 2 g/L glucose. After 48 h, the medium was collected by centrifugation to remove the cells. Latate in the supernatant was detected according to the manufacturer's instructions. Spectrophotometric absorbance was measured at 530 nm using a multi-plate reader (Multiskan Spectrum). Values were obtained by comparing with a lactate standard solution.

### Western blotting analysis

Western blotting analysis was performed as described previously [19]. Briefly, cells (10^6^) were washed twice in cold PBS and then lysed by Laemmli sample buffer (Bio-Rad). Samples were boiled for 5 min at 100°C. Proteins were separated by 8%~15% SDS–polyacrylamide gel electrophoresis and transferred onto nitrocellulose membranes (0.22 μm or 0.45 μm, Millipore). Non-specific binding sites were blocked with 5% non-fat dry milk dissolved in TBS (10 mM Tris-HCl, pH 7.6, 137 mM NaCl) with 0.1% Tween 20 (TTBS) for 2 h at room temperature, followed by incubation with primary antibody for 2 h at room temperature or at 4°C overnight. The membranes were then washed 3 times in TTBS and incubated for 2 h at room temperature with secondary HRP–conjugated donkey anti-rabbit antibody or HRP-conjugated sheep anti-mouse antibody diluted 1:5000 in TTBS with 5% non-fat milk. Proteins were visualized by incubation with ECL plus (Millipore). All experiments were carried out independently at least 3 times. The level of β-Actin protein was used as a control for the amount of protein loaded into each lane.

### Statistical analysis

All assays were performed in triplicate, and data are expressed as mean values ± SD. One-way ANOVA was used to compare two groups. A *p-value* < 0.05 was considered to be significant.

## Abbreviations

ALL: acute lymphoblastic leukemia
AMPK: AMP-activated protein kinase
ATP: adenosine triphosphate
Dex: dexamethasone
ER: endoplasmic reticulum
HKII: hexokinase II
GC: glucocorticoid
p70S6K: p70S6 kinase
p-p70S6K: p70S6K phosphorylation
2-DG: 2-deoxy-D-glucose
